# A Novel Key Distribution for Mobile Patient Authentication Inspired by the Federated Learning Concept and Based on the Diffie–Hellman Elliptic Curve

**DOI:** 10.3390/s25082357

**Published:** 2025-04-08

**Authors:** Orieb AbuAlghanam, Hadeel Alazzam, Wesam Almobaideen, Maha Saadeh, Heba Saadeh

**Affiliations:** 1 Department of Computer Science, The University of Jordan, Amman 77110, Jordan; wxacad@rit.edu (W.A.); heba.saadeh@ju.edu.jo (H.S.); 2 Department of Information Technology, Yarmouk University, Irbid 21110, Jordan; hadeel.alazzam@yu.edu.jo; 3 Department of Electrical Engineering and Computing Sciences, Rochester Institute of Technology, Dubai P.O. Box 341055, United Arab Emirates; 4 Department of Computer Engineering and Informatics, Middlesex University Dubai, Dubai P.O. Box 500697, United Arab Emirates; m.saadeh@mdx.ac.ae

**Keywords:** authentication, AVISPA, BAN logic, e-healthcare system, federated learning (FL)

## Abstract

Ensuring secure communication for mobile patients in e-healthcare requires an efficient and robust key distribution mechanism. This study introduces a novel hierarchical key distribution architecture inspired by federated learning (FL), enabling seamless authentication for patients moving across different healthcare centers. Unlike existing approaches, the proposed system allows a central healthcare authority to share global security parameters with subordinate units, which then combine these with their own local parameters to generate and distribute symmetric keys to mobile patients. This FL-inspired method ensures that patients only need to store a single key, significantly reducing storage overhead while maintaining security. The architecture was rigorously evaluated using SPAN-AVISPA for formal security verification and BAN logic for authentication protocol analysis. Performance metrics—including storage, computation, and communication costs—were assessed, demonstrating that the system minimizes the computational load and reduces the number of exchanged messages during authentication compared to traditional methods. By leveraging FL principles, the solution enhances scalability and efficiency, particularly in dynamic healthcare environments where patients frequently switch between facilities. This work bridges a critical gap in e-healthcare security, offering a lightweight, scalable, and secure key distribution framework tailored for mobile patient authentication.

## 1. Introduction

In the age of interconnected healthcare systems, the Internet of Medical Things (IoMT) has emerged as a transformative force, revolutionizing the way healthcare data are collected, analyzed, and used [1]. From wearable devices that monitor vital signs to smart implants that transmit real-time health information, IoMT has ushered in an era of unprecedented medical data generation [2]. However, with this abundance of data comes a pressing need for robust security and authentication mechanisms to ensure the integrity and privacy of patient information.

The benefits of IoMT are indeed profound. It enables remote patient monitoring, facilitating the continuous collection of health data outside traditional clinical settings [3]. This empowers healthcare providers to offer personalized and proactive care, detect health problems early, and make data-driven decisions, ultimately improving patient outcomes. In addition, IoMT improves the efficiency of medical services, reducing the burden on healthcare facilities by enabling telemedicine, remote consultations, and even the possibility of timely interventions through predictive analytics [4].

At the same time, alongside these remarkable advantages, IoMT introduces a series of complex challenges. Security and privacy vulnerabilities are large as data flows between numerous devices, networks, and cloud platforms [5]. Unauthorized access to medical data poses serious risks, making robust authentication mechanisms paramount. Interoperability issues also arise as various devices and platforms must communicate and share data seamlessly while maintaining data integrity [6].

In this intricate landscape, the need for secure and efficient patient authentication mechanisms becomes increasingly evident. Ensuring that healthcare providers have access to the right patient’s data is not just a matter of convenience, but a fundamental requirement to provide safe and effective care [7]. Traditional methods of patient authentication, often relying on static identifiers such as usernames and passwords, are not suited to the dynamic and interconnected world of IoMT. They leave room for vulnerabilities and may hinder the full realization of IoMT’s potential [8].

Patient authentication within the IoMT ecosystem is a multifaceted challenge with far-reaching implications. It encompasses the methods and technologies used to verify the identity of patients and healthcare providers who access IoMT devices and the data they generate [9]. The fundamental goals of patient authentication in IoMT are twofold: to ensure the security and integrity of medical data and to protect patient privacy [10].

IoMT introduces unique challenges to patient authentication. The diverse array of devices, ranging from wearable sensors to implantable medical devices, requires authentication methods that can accommodate various form factors and communication protocols. These devices must seamlessly integrate into the broader healthcare infrastructure while ensuring the privacy and security of patient data [11].

Additionally, the real-time nature of IoMT data transmission necessitates authentication mechanisms that can operate swiftly and efficiently, without causing delays in medical data access or decision-making processes. Balancing security, speed, and usability is paramount in the IoMT environment [12].

One critical aspect of securing data transmitted within the IoMT ecosystem is the use of robust encryption techniques [10]. Encryption ensures that data are protected from unauthorized access while in transit. However, effective encryption relies on the secure distribution of encryption keys.

Key distribution in the context of IoMT involves the secure exchange and management of cryptographic keys between devices, users, and healthcare systems. These keys are essential for encrypting and decrypting data as they move between IoMT devices, ensuring that sensitive medical information remains confidential and tamper-proof during transmission.

Establishing a secure and efficient key distribution system is paramount for the overall data security within IoMT [13]. It involves addressing challenges such as key management, key generation, and key revocation, while ensuring that patient authentication seamlessly integrates with the encryption process [14].

**Federated Learning (FL)** is a collaborative, decentralized, and distributed iterative procedure in which individuals work together to train machine learning models while protecting the confidentiality of their personal data [15]. This approach is exemplified by its implementation in Google Gboard [16]. Rather than transmitting raw data, terminal devices (clients) locally process their information and then forward only the modifications to a central system (server). Subsequently, the server compiles and aggregates the modifications from all clients to generate an updated global model, which is then redistributed to the clients [17]. This iterative process persists until an optimal global model is achieved allowing for continuous improvement of the global model without exposing sensitive data to a centralized entity, making federated learning a promising technique for training machine learning models in privacy-sensitive scenarios [18]. FL has applications in various domains, such as healthcare, finance, Internet of Things (IoT), and edge computing, where data privacy, network bandwidth limitations, or regulatory constraints make centralized training impractical or undesirable [19,20].

A privacy-preserving domain refers to an area or context where techniques, methods, or systems are employed to protect and maintain the privacy of individuals’ sensitive information. In such a domain, measures are taken to ensure that personal data are securely handled and are used in a way that minimizes the risk of unauthorized access, disclosure, or misuse [21,22]. This can be achieved using different approaches such as data anonymization, encryption, differential privacy, access controls, secure multi-party computation (SMPC), and FL [23].

**Figure 1:**
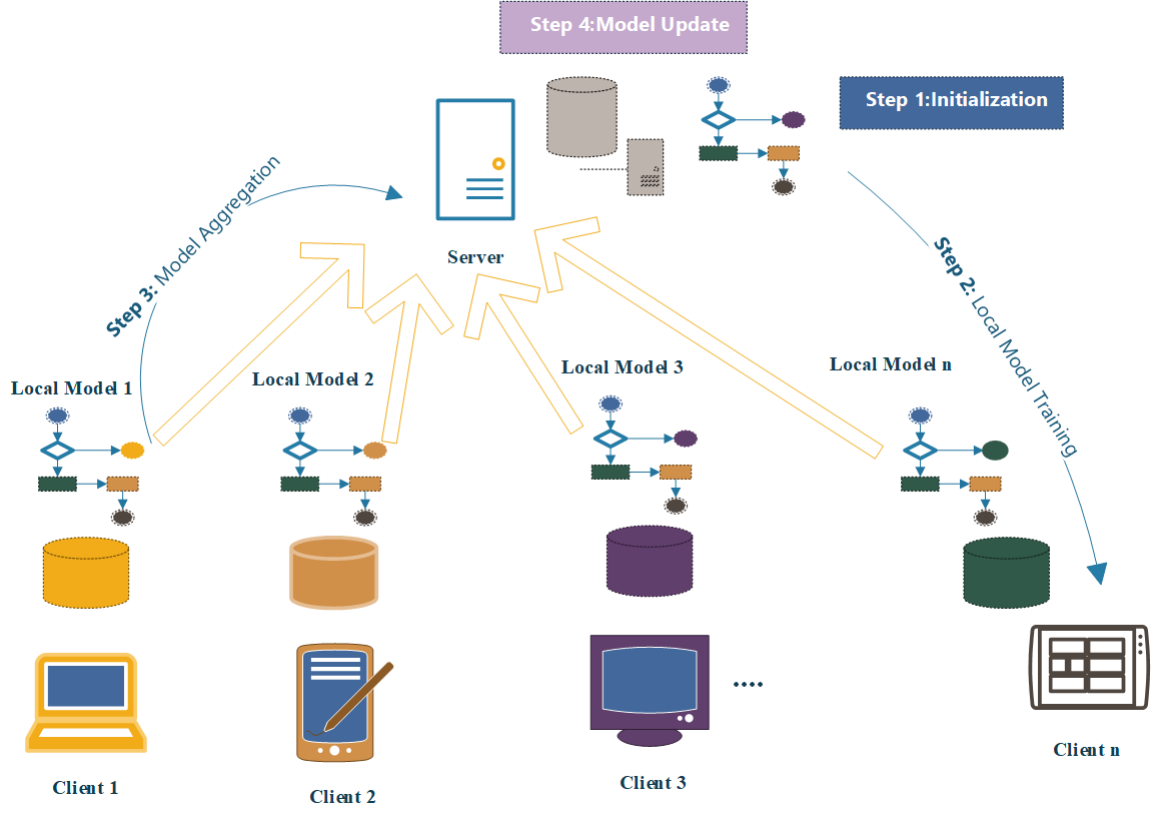
Federated learning steps.

The process of FL passes through different steps as illustrates in Figure 1. It begins with the Initialization Phase, where a central server initializes a global model and distributes it to participating devices or servers. In the subsequent local model training phase, each device trains the model independently using its local data, safeguarding the raw data’s privacy [24]. The updated models’ parameters are then transmitted back to the central server during the model aggregation phase, where they are combined from all participating devices or servers [25]. Following this, the central server performs a model update, incorporating the aggregated parameters into the global model. The updated global model is subsequently sent back to the participating devices, commencing the next round of local training [26,27]. This iterative process, including local training, model aggregation, and model updates, continues until the desired level of model performance is achieved or convergence is reached [15,27,28].

### 1.1. Challenges to Ensuring Authentication and Confidentiality in Healthcare

Securing healthcare systems is a critical endeavor, but it comes with several challenges such as data privacy, cybersecurity threats, interoperability, mobile and IoT devices, human errors, legacy systems, resource constraints, emerging technologies, patient mobility, and regulatory compliance [29].

Ensuring security in various applications, particularly in healthcare systems, has become a prominent area of research. This entails addressing multiple security facets, including confidentiality, authentication, and privacy. Preserving patient privacy is of the utmost importance. Establishing a secure connection through data encryption between the sender and receiver is crucial. Moreover, accommodating patient mobility is a significant consideration [30,31].

In this paper, we will focus on three challenges: patient mobility, privacy, and resource constraints.

### 1.2. Contributions

1. Proposing a hierarchical key distribution architecture in the context of an e-healthcare system to enhance the security and privacy of patients based on FL.
2. Proposing a novel key distribution protocol based on FL to exchange the local and global model between the root public key generator $Root_{PKG}$ and sub-public key generator $Sub_{PKG}$.
3. Designing a lightweight key establishing approach between $Sub_{PKG}$ and mobile patient using Diffie–Hellman elliptic curve algorithm.
4. Providing patient authentication using a private key and several secure parameters also providing identity preservation using the concept of a local model for each $Sub_{PKG}$.
5. Enhancing the performance for the mobile node in terms of the number of exchange messages [32].

Our work primarily follows a scientific approach, as it focuses on enhancing the security and privacy of patients by proposing a hierarchical key distribution architecture in the context of an e-healthcare system with simulation and formal security proofs.

## 2. Related Work

This section provides a summary of the authentication techniques discussed in the literature, as illustrated in Table 1. It highlights key methodologies, their effectiveness, and the contexts in which they are applied. Additionally, a comparative analysis is presented to examine the authentication techniques and the environments for which they are designed.

Table 2 provides a comparative analysis of different healthcare systems discussed in the literature. The comparisons have been performed using various architectures, security goals, verification methods, and performance aspects, offering insights into their strengths and limitations.

Many authentication techniques have been used in the literature. For instance, the authors of [33] employ fingerprint recognition as a biometric modality and extract specific features to create a shared cryptographic key. Their proposed methodology includes three phases: System Initialization, Patient Registration, and Mutual Authentication with Session Key Agreement. The System Initialization Phase involves two key steps: extracting a cancelable biometric template and generating system parameters. The authors of [34] propose a lightweight and robust authentication scheme utilizing a simple hash cryptographic function, with public–private key pairs designed specifically for IoMT devices. They utilize formal analysis techniques like BAN logic and ProVerif2.02, in conjunction with informal pragmatic illustration, to validate the effectiveness of their proposed protocol. Moreover, the study conducts a performance analysis to showcase the delicate balance achieved between security and efficiency, an aspect frequently overlooked in existing solutions.

Another study that uses biometric data for patient authentication is [35]. The authors propose a framework that integrates wearable sensors to monitor vital signs and authenticate patients using biometric data alongside traditional credentials, secured by the SHA-512 algorithm. Sensor data transmission to the cloud is encrypted with the Substitution-Ceaser cipher and improved Elliptical Curve Cryptography (IECC), with enhanced security from an additional secret key. Despite increased complexity, the approach remains computationally efficient, with encryption and decryption times of 1.032 μ and 1.004 μ, respectively, and performance analysis shows strong algorithm reliability compared to RSA and ECC.

The authors of [36] introduce a lightweight anonymous mutual authentication and key agreement scheme for Wireless Body Area Networks (WBANs). This scheme relies solely on hash function operations and XOR operations. The authors employ the automatic security verification tool ProVerif to verify the security properties of their scheme, complemented by informal security analysis. Furthermore, they conduct a comparative analysis of their proposed scheme against several related works. The results demonstrate that their scheme either offers superior advantages in terms of computation cost, energy consumption, and communication cost, or presents lower security risks compared to existing approaches.

Alzahrani et al. [37] present a review of the patient healthcare monitoring and authentication protocol designed for Wireless Body Area Network (WBAN) environments proposed by Xu et al. in [36]. While Xu et al.’s scheme demonstrates efficiency in terms of computation by employing lightweight operations, the conducted analysis uncovers several security loopholes. It is revealed that Xu et al.’s protocol is susceptible to various threats, including replay attacks, key compromise impersonation (KCI) attacks, and privacy concerns. In response to these vulnerabilities, they propose a new authenticated key agreement protocol tailored for WBANs. The security properties of the improved protocol are formally verified and validated through BAN logic analysis and the ProVerif automated simulation tool.

**Table 1:** Mobile patient authentication in the literature.

Reference	Authentication Technique	Environment
[38]	Biometric+ECC	Mobile healthcare environments
[34]	Shared key	Wireless Medical Sensor Networks
[35]	ECC	IoT
[37]	Improved mutual authentication	Wireless Body Area Networks
[36]	Lightweight anonymous mutual authentication	Wireless Body Area Networks
[39]	Federated learning and blockchain	IoT healthcare
Our proposal	Federated learning and key distribution	IoT healthcare system

The authors of [38] propose a secure and lightweight remote patient authentication scheme tailored for mobile healthcare settings. Their approach translates patient biometric data into Elliptic Curve Cryptography (ECC)-based keys, enabling secure and cost-effective authentication without the need to store or transmit biometric templates. Moreover, the proposed approach provides mutual authentication with session key agreement and resists various types of attacks. Singh et al. in [39] explore the applications of federated learning in establishing a distributed secure environment within smart cities. In addition, they propose a secure architecture for privacy preserving in smart healthcare, utilizing blockchain and FL technologies. Blockchain-based IoT cloud platforms enhance security and privacy, while FL enables scalable machine learning applications, particularly in healthcare. Importantly, users can access well-trained machine learning models without compromising personal data through federated learning.

**Table 2:** Different healthcare systems in the literature.

Reference	Architecture	Security Goal	Verification Method	Performance Analysis
[40]	Cloud of things	Prevent Man-in-the-Middle (MITM)	Scyther	Anonymity, Authentication, Authorization, Accountability, Confidentiality, Integrity, Non-repudiation
[41]	IoT-based M-Health system	Signature, Encryption, and Signcryption	Mathematical proof	Computational Cost, Communication Cost
[42]	WBANs	Authentication	Mathematical proof	Storage Overhead, Computation Cost, Communication Cost
[43]	E-healthcare	Authentication	AVISPA and BAN logic	Storage Cost, Communication Cost, Computation Cost
Our proposal	IoT healthcare	Authentication, Confidentiality, and Privacy	AVISPA and BAN logic	Storage Cost, Communication Cost, Computation Cost

## 3. Proposed System

### 3.1. Architecture Overview

**Figure 2:**
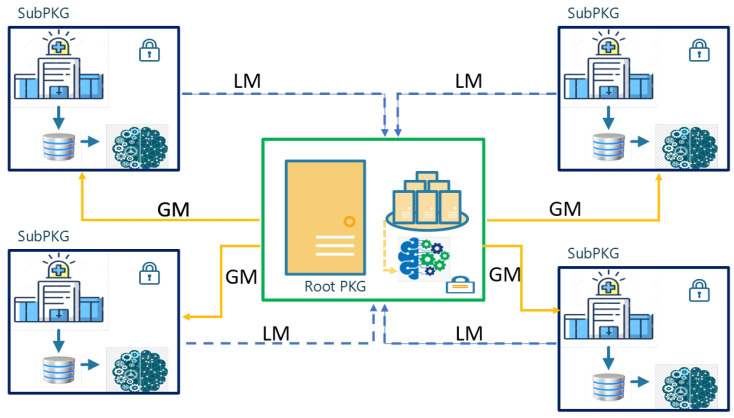
Hierarchy architecture for key distribution using federated learning.

This section proposes a key distribution architecture inspired by FL, as illustrated in Figure 2. Additionally, Table 3 presents the abbreviations used in this paper. The architecture consists of Root PKG, several Sub-PKGs, and patients. In each Sub-PKG, there is a local model to keep the data for each patient who belongs to this party. In the Root PKG, there is a global model that handle all local models without having knowledge about the patients in each Sub-PKG. In this architecture, several challenges have been addressed, such as computational complexity, communication demands, and storage requirements for mobile patients. Additionally, it prioritizes the secure key distribution process to safeguard patient privacy, even in situations where patients change their positions, all while minimizing unnecessary complexity.

It can be noticed that the Root PKG has a global model that aims to distribute common parameters for all Sub-PKGs based on the received local models from Sub-PKGs. Moreover, each Sub-PKG authenticates the patient using a shared common key between them. Thus, any mobile patient moving to another Sub-PKG can be authenticated without asking the original Sub-PKG.

In this architecture, we will employ the concept of FL to design a lightweight protocol to provide a secure connection between the patient and the hospital. Thus, the patient can establish a shared key using Elliptic Curve Diffie–Hellman (ECDH) and can be authenticated by the other parts (Sub-PKGs). ECDH is a cryptographic key exchange and authentication protocol commonly used to establish a secure communication channel between two parties over an insecure network [44].

### 3.2. The Proposed Protocol Overview

The proposed protocol consists of three phases: the first phase, called the Initialization Phase, between $Root_{PKG}$ and $Sub_{PKG}$. In the second phase, the patient registers for an official $Sub_{PKG}$. The third phase is the Mobile Patient Authentication Phase, which allows an easy way for any mobile patient node to access any $Sub_{PKG}$ without returning to the old one. On the other hand, further details on Elliptic Curve Diffie–Hellman can be found in Appendix A.1. The following subsections discuss each phase in detail.

#### 3.2.1. Initialization Phase Between $Root_{PKG}$ and $Sub_{PKG}$

**Figure 3:**
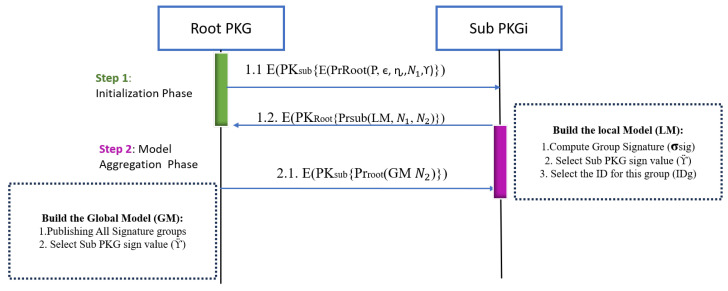
The proposed protocol at Initialization Phase.

During this phase, the $Root_{PKG}$ initializes and distributes specific set of parameters for each $Sub_{PKG}$ to assist them in creating their local models. After each $Sub_{PKG}$ has finished building its local model, it sends its local model back to the Root PKG. The Root PKG, in turn, utilizes these local models to combine them into the ultimate model, often known as the global model. Figure 3 illustrates the process of the Initialization Phase, which consists of a two-step Initialization Phase and the model aggregation phase.

Each $Sub_{PKG}$ has its own public key and private key, while the same applies to the $Root_{PKG}$. $Root_{PKG}$ is considered as one trusted point for all other $Sub_{PKG}$s. Thus, for any further connection between any $Sub_{PKG}$s, they should exchange their digital signature which is represented in Equation (1):

(1) $$DC{\left(SubPKGi\right)}_{\left(root,sub_{i}\right)}=\left\{\begin{array}{c} E_{Pr_{root},\left(PK_{sub_{i}},ID_{sub},T^{\prime }\right)} \end{array}\right.$$

The following steps conclude the details of each connection:

1. $Root_{PKG}$ initiates a request to send information about the whole system, which consists of all Sub-PKGs and patients. This information is essential for the Sub-PKGs to construct their local models while considering these parameters.
2. Root parameters are elliptic curve ranges for each Sub-PKG (ϵ), ranges for group signature (η), and root sign value (γ).
3. The parameters are securely handled. Initially, ensuring nonrejection properties, the $Root_{PKG}$ encrypts these parameters using its private key, to ensure integrity and authentication. Subsequently, it encrypts them once more using the public key provided by the respective Sub-PKG to maintain confidentiality.
4. After each $Sub_{PKG}$ receives the parameters from the root and based on the number of patients that it needs to deal with, the $Sub_{PKG}$ determines the group signature $\sigma_{sig}$, Sub-PKG signing value Υ, and identity for the group $ID_{g}$. After that, the $Sub_{PKG}$ sends a local model via its private key and encapsulates via the root public key.
5. The root aggregates multiple local models from various Sub-PKGs, each equipped with its own set of parameters. This collective information is then used to construct the global model, ensuring a comprehensive consideration of all these individual parameters.

#### 3.2.2. Patient Registration and Key Generation Phase

**Figure 4:**
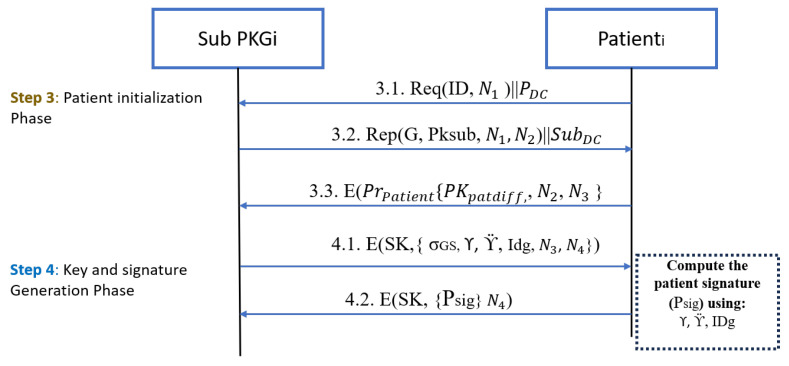
Patient Registration Phase.

Figure 4 illustrates this phase; each patient is assigned to their respective $Sub_{PKG}$. They must agree on various parameters and establish a shared key. This shared key will then be utilized for future connections. The $Sub_{PKG}$ needs to authenticate patients. When the patient asks for the $Sub_{PKG}$ for the first time for registration to be part of a group, the patient must show the $Sub_{PKG}$ acceptable credentials.

The $Sub_{PKG}$ thoroughly reviews it and checks whether anything about the patient is suspicious. If the $Sub_{PKG}$ is fully satisfied with the background verification, it accepts the patient’s request to be part of the group. The $Sub_{PKG}$ and the patient create a shared key using **ECDH** as follows:

1. The $Sub_{PKG}$ selects generator G based on the range that was created by the $Root_{PKG}$, prime number p, and selects a private number $d_{sub}$ to determine the pubic key as $Psub=G^{d_{sub}}modp$.
2. The patient selects the private key $d_{patient}$ then determines its public key as $Ppatient=G^{d_{patient}}modp$ and sends the public key to the $Sub_{PKG}$. Moreover, it determines the shared key as $SK=Psub*d_{patient}$.
3. After the $Sub_{PKG}$ receives the patient’s public key it will determine the shared key as $SK=Ppatient*d_{sub}$.

After the shared key has been established between the patient and the $Sub_{PKG}$, the $Sub_{PKG}$ sends an encrypted message using the shared key that holds the group signature ($\sigma_{GS}$), the identity of the group ($ID_{g}$), the root sign (γ), and the Sub-PKG sign (Υ).

The patient computes their own signature after receiving the parameters from $Sub_{PKG}$ using ECDSA Sign based on the following points:

1. Compute a message digest of the data you want to sign, often using a cryptographic hash function like SHA-256.
2. The patient computes the digest for the message that equals $\sigma_{sig}+\Upsilon +\gamma$; this will be as one block while ϰ is unique for each patient.
  (2) $$Message=\sigma_{sig}+\Upsilon +\gamma +\varkappa$$
3. Generate a random number k in the range [1, n−1].
4. Compute the point ($X_{1}$,$Y_{1}$) = k*G.
5. Calculate r = $X_{1}$ mod n.
6. Calculate s = $\frac{H\left(M\right)+d_{patient}*r}{K}$mod n.
7. The patient ’s signature ($P_{sig}$) is (r + ϰ,s + ϰ).

#### 3.2.3. Mobile Patient Authentication

**Figure 5:**
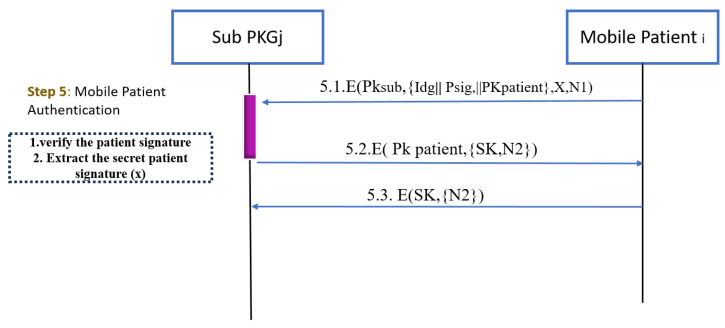
The mobile Patient Authentication Phase.

Figure 5 illustrates the steps that are needed for any mobile patient that needs to change its location. The bottleneck in [32] is that if any mobile moves to a different Sub-PKG domain, it needs around nine messages to distribute a shared key, and the mobile node should ask the gateway. In this protocol, the patient has a signature and a group identity. Once the patient changes their location and wants to join another Sub-PKG domain, the following procedure is applied.

For verification, the Sub-PKG tries first to extract the patient secret value **ϰ** as follows:

1. A mobile patient sends a request for $Sub_{PKGj}$ to obtain authentication and to be allowed to enter this domain.
2. To achieve confidentiality, the mobile patient encrypts its request via $Sub_{PKGj}$’s public key.
3. The request contains the patient signature, patient public key, group identity, hashed secret value, and nonce.
4. $Sub_{PKGj}$ extracts the ϰ assuming that the Sub-PKG has initial values for each $\sigma_{sig},\Upsilon ,and\gamma$ that exist in the global model. $x=M-(\sigma_{sig}+\Upsilon +\gamma$) then checks this value by hashing it then compares it with the received digest. After that, it hashes the message using hash algorithm h = H(M).
5. $Sub_{PKGj}$ find the exact value of (r,s) after subtracting the patient’s secret value from each ϰ.
6. Calculate the modular inverse of the signature proof $s1=s^{\left(-1\right)}modn$.
7. Recover the random point used during the signing: R’ = (h ∗ s1) ∗ G + (r ∗ s1) ∗ pubKey.
8. Take from R’ its x-coordinate: r’ = R’.x.
9. Calculate the signature validation result by comparing whether r’ == r.

## 4. Security Analysis

### 4.1. Security Proof Using AVISPA Tool

We conduct a comprehensive security verification using Automated Validation of Internet Security Protocols and Applications (AVISPA), which yields outcomes aligned with the protocol’s objectives. Initially scripted in CAS+, the protocol is subsequently translated into HLPSL code to scrutinize the integrity and confidentiality of crucial components such as keys, signatures, and confidential messages. As an illustration of our methodology, we instantiate three entities: $Root_{pkg}$, $Sub_{pkg}$, and the mobile patient. In the first phase, we focus on the authentication between $Root_{pkg}$ and $Sub_{pkg}$ as well as the secrecy of the local model of the $Sub_{pkg}$. The next two phases in the proposed protocol are the Patient Authentication Phase and the Patient Registration Phase. The results are simulated and indicate that the protocol is safe against attacks, see Appendix A.2.

### 4.2. BAN Logic

BAN logic [45,46] is used to formally verify the proposed protocol. BAN logic is a logic of authentication proposed by Burrows, Abadi, and Needham [45]. It uses inference rules and it is based on some initial assumptions to infer new facts that can lead to the aims being achieved. BAN logic has been used to analyze the security of authentication protocols against some of the most common attacks, like the Man-in-the-Middle attack, Intercept-and-Resend attack, and replay attack [47]. The proposed authentication protocol is verified using BAN logic, since it is useful to verify such protocols, which are based on fresh values and trust [48]. For further details regards BAN logic rules and notations, please refer to [45,49]. The full proof and analysis of the BAN logic are included in Appendix A.3.

### 4.3. Security Analysis Against Well-Known Attack

This section discussed the threat model and how the proposed scheme is secure against the most common attacks.

- **Brute Force Attack:** The attacker cannot reveal the private keys or the symmetric keys in a reasonable time. The EC private key cannot be calculated from the EC public key since it is an Elliptic Curve Discrete Logarithm Problem (ECDLP) [49,50]. The session key SK is generated using the ECDH algorithm which is based on the use of an EC private key for either the patient node or the SubPKG. Breaking ECDH session keys requires solving ECDLP, which is infeasible with classical computers. Consequently, brute force attacks will fail.
- **Man-In-The Middle and Eavesdropping Attack:** The attacker can intercept the communication between the patient’s node and the $Sub_{PKG}$ to read the data shared between these two entities; however, the attack will fail. In the Patient Authentication Phase, all messages are encrypted either using ECC to encrypt message 5.1 with the $Sub_{PKG}$ public key, or ECDH to encrypt messages 5.2 and 5.3 with the symmetric key SK. To decrypt the messages, the attacker should have the $Sub_{PKG}$ private key which is known only to the $Sub_{PKG}$ and the symmetric key SK which is known only to the patient node and the $Sub_{PKG}$. Consequently, this attack will fail.
- **Replay Attack:** The attacker will try to perform a replay attack by resending a valid message to the $Sub_{PKG}$; however, the $Sub_{PKG}$ can detect this attack. Replay attacks can be detected using nonce values. In the Patient Authentication Phase, whenever the patient node should be authenticated, a nonce N1 is generated at the node side and passed to the $Sub_{PKG}$. Another nonce N2 is also generated at the $Sub_{PKG}$ side and passed to the node. By verifying the freshness of these nonce values, both the patient’s node and the $Sub_{PKG}$ can detect the replay attack. A similar approach is used to detect replay attacks in the Patient Registration Phase; when the signature is calculated, a nonce value is generated by the node and passed to the $Sub_{PKG}$ along with the signature. By verifying the freshness of the nonce value, the $Sub_{PKG}$ will make sure that the signature is newly generated; if not, a replay attack is detected due to nonce verification failure.
- **Signature Forgery Attack:** The attacker tries to impersonate the patient’s node by forging the patient’s node signature; however, this attack will fail. In order to forge a signature, the attacker needs to know the group signature ($\sigma_{GS}$), the identity of the group ($ID_{g}$), the root sign (γ), and the Sub-PKG sign (Υ). These parameters are shared by the $Sub_{PKG}$ in an encrypted message during the Patient Registration Phase. The message is encrypted using a session key SK, which is known only to the patient node and the $Sub_{PKG}$; consequently, the attacker will not be able to know the parameters. In addition to these parameters, the attacker should know the patient node’s private key, which is known only to the node.
- **Unauthorized Access and Identity Theft:** If the attacker gains access to a patient’s signature, they could impersonate a patient and access personal health information. To mitigate this threat, all communication messages between the patient’s node and the $Sub_{PKG}$ are encrypted. Consequently, the attacker will not be able to access the patient’s signature.

## 5. Experimental Results and Discussion

In this section, a deeper analysis of the proposed protocol’s complexity in terms of storage, computation, and communication cost is presented. Moreover, a comparison with other related works in terms of several performance metrics is conducted.

### 5.1. Storage Cost

The storage cost refers to the amount of memory required to store the parameters necessary for establishing a shared key for the **mobile patient node**. Furthermore, the incurred cost varies based on the specific type of parameter stored within the mobile node.

The **Total Storage Cost (TSC)** is measured in bits by using the default sizes for each of them. The size of the symmetric key is fixed to 128 bits, and the identifier and the group signatures are saved in 256 bits for the mobile patient node. The public key size is 256 bits assuming that NIST P-256 curve (secp256r1) has been used [51].

In the proposed architecture, we focus on reducing the memory cost of the mobile node in terms of the number of keys and the number of initial parameters that each node should have. The following equation shows the exact complexity for the mobile patient node.

Table 4 presents the size of each parameter that has been used in this paper. The digital signature that has been used in the proposed protocol is based on ECDSA and NIST P-256 curve where r and s have 32 bytes. Therefore, the digital signature’s size is 64 bytes, while the public key and the private key have 64 bytes. Unique Identifiers (UIDs: 16 bytes) are used to identify each patient node.

Elliptic Curve Diffie–Hellman (ECDH) is used, so the size of the shared key is 32 bytes. The root and patient signature have 64 bytes while there is 64 bytes for the patient’s secret value [52,53,54]. Table 5 presents the storage complexity for each node in our architecture. It can be seen that the main objective of the proposed protocol is to reduce the storage complexity of the patient node due to the fact that each $Root_{PKG}$ and SubPKG are considered powerful devices.

Table 6 presents a comparative study in terms of the memory storage costs required by the mobile nodes and different related protocols. The authors of [55] propose a new and improved group signature scheme base on federated learning. The main goal of their proposal is to reduce the commutation and computation cost to provide efficient privacy. In [56], a full dynamic secret sharing is proposed for federated learning. It can be noticed that our approach outperforms the others due to the fact that only one shared key is stored in the patient node. In [57], a Remote Authentication Method (RAM) with Autonomous Shared Keys (ASK) is introduced in the context of medical applications.

### 5.2. Computation Cost

The proposed scheme aims to authenticate the patient’s mobile node based on the computed patient signature. The computation cost analysis considers the patient signature generation cost $P_{sig}$, which is generated once in the Patient Registration Phase and reused in the Patient Authentication Phase, and the patient signature verification cost which is performed by the $Sub_{PKG}$ in the Patient Registration Phase and the Patient Authentication Phase. As discussed in Section 3.2.2, signature generation and verification are based on ECDSA. Table 7 presents the notations used to measure the computation cost.

**ECDSA Analysis:** The cost and efficiency of ECDSA schemes depend on the number of operations used in the methods. Let TM represent the computing time required for the modular multiplication operation, TA the computing time required for the modular addition operation, and TIN the computing time required for the modular inversion operation. TEM represents the computing time required for the elliptic curve multiplication operation. Finally, TEA is the computing time required for the elliptic curve addition operation. According to the literature [58,59], TA is much less than TM and can be negligible. Assuming that TM is the main operation, the computation cost of TIN, TEM, and TEA in terms of modular multiplication operation TM can be calculated as follows: TIN = 11.6 TM, TEM = 29 TM, and TEA = 0.12 TM [58,59,60].

According to the computation cost required for different operations discussed above, we can analyze the computation cost of the ECDSA signature generation and verification as follows: To generate the signature on the mobile patient node, ECDSA requires one modular multiplication operation, one elliptic curve multiplication operation, and one modular inversion operation which equals TM + TEM + TIN = 41.6 TM. To verify the signature on the $Sub_{PKG}$ side, ECDSA requires two modular multiplication operations, two elliptic curve multiplication operations, one modular inversion operation, and one elliptic curve addition operation which equals 2TM + 2TEM + TIN + TEA = 71.72 TM. Note that, in our calculation, the modular addition operation is ignored as it is negligible [58,59].

Table 8 shows the computation cost compared with other signature-based authentication schemes from the literature. Note that n indicates whenever a node needs to be authenticated. The main advantage of our scheme is that even the signature generation cost is slightly higher than some related schemes; however, it is computed once on the mobile patient’s node. On the other hand, in the other schemes, the signature is generated every time a node/client should be authenticated.

### 5.3. Communication Cost

**Figure 6:**
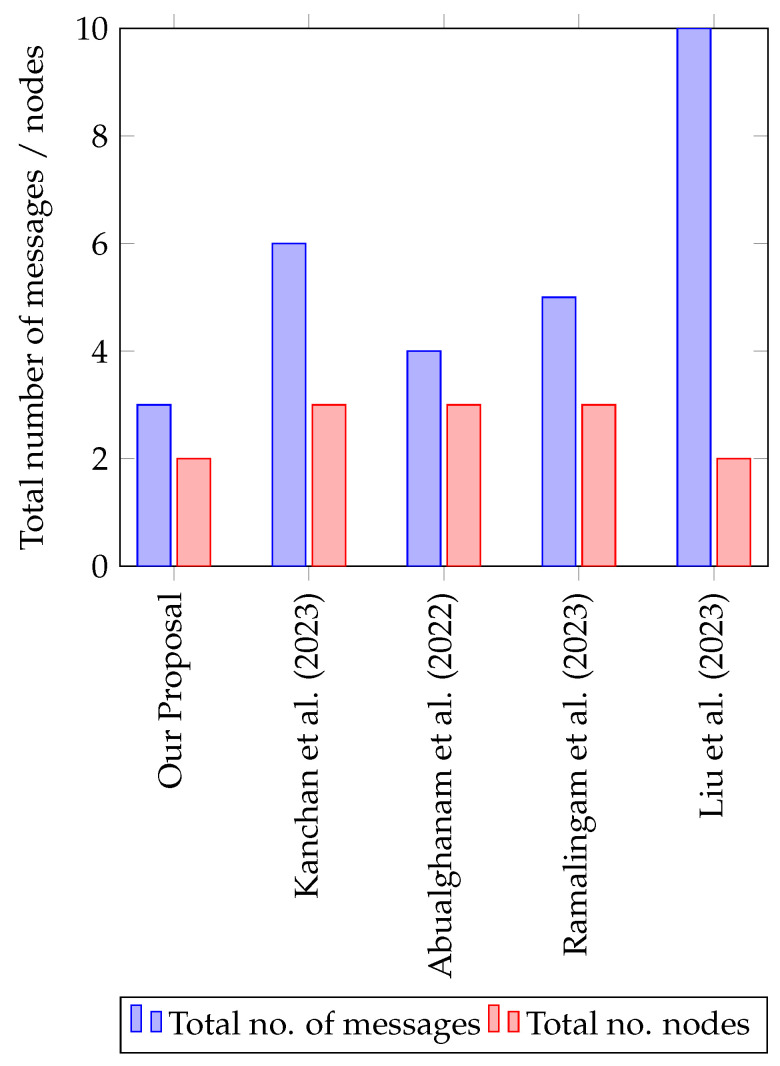
The communication cost of various protocols for the mobile patient node. The references are: Kanchan et al. (2023) [55], Abualghanam et al. (2022) [32], Ramalingam et al. (2023) [57], and Liu et al. (2023) [56].

The communication cost in any network refers to the resources consumed during data transmission between devices or nodes [61]. In this paper, the number of exchanged messages required for any mobile patient node to be authenticated by its associated $Sub_{PKG}$ is analyzed. Figure 6 illustrates the differences in communication costs across various protocols for the mobile patient node in terms of the total number of messages and the number of nodes that should work together in order to distribute the key. It can be noticed that our proposed method demonstrates a lower communication overhead compared to the other related proposals.

In [56], the highest number of messages is shown for distributing the key between two nodes. In [32], the proposed approach maintains a moderate balance between the number of messages and nodes, while [57] shows a noticeable increase in total messages, though the number of nodes remains controlled. In contrast, our proposal effectively reduces both message exchanges and node involvement, enhancing efficiency in mobile environments.

## 6. Conclusions

Maintaining privacy and security is vital in healthcare systems, especially those that support mobile patients who need to be authenticated and protected while on the move. This study investigated the integration of the concept inspired by federated learning and the hierarchical structure of public key generators used in layered healthcare systems to authenticate different mobile patients. Both the BAN logic and the SPAN-AVISPA tool were used to verify the validity of the proposed architecture and the performance of key distribution protocols designed based on it. In addition, the storage requirement and computation and communication costs of the mobile patient nodes were used to assess the performance of the proposed protocol. The effectiveness of the proposed key distribution protocol outperformed other similar protocols in terms of reducing computation and communication costs. In addition, it reduced the number of messages needed for an authentication protocol and the number of shared keys that must be exchanged and stored at each patient’s mobile node to only one key.

## Figures and Tables

**Table 3:** List of symbols and abbreviations used in the paper for notation and computation purposes.

Notations	Description
GM	Global model
LM	Local model
PKG	Public key generator
$PK_{Root}$	Public key for the $Root_{PKG}$
$Pr_{Root}$	Private key for the $Root_{PKG}$
$DC_{Root}$	Digital signature for $Root_{PKG}$
$DC_{Sub}$	Digital signature for $Sub_{PKG}$
ID	Real identity of the patient
H	Hash function
ϵ	Elliptic curve range
η	Ranges for group signature
γ	Root sign value
Υ	Sub-PKG sign value

**Table 4:** The size in byte for each abbreviation in the proposed protocol.

Abbreviation	Size (Byte)
Digital Signature	64
Public Key	64
Private Key	64
Unique Identifiers	16
Shared Key	32
Patient Signature	64
Root Signature (γ)	64
Patient Secret Value (ϰ)	64

**Table 5:** The storage complexity for each node in the proposed protocols.

Node Type	Storage	Size in (Bytes)
$Root_{PKG}$	$PK_{Root}$, $Pr_{Root}$, $DC_{Root}$, root parameters ϵ, η, γ, n*Υ.	384 + 64 n
$Sub_{PKG}$	$PK_{Sub}$, $Pr_{Sub}$, $DC_{Sub}$, m*SK, γ, Υ	320 + 32 m
$Mobile_{Patient}$	SK, $Id_{i}$, $P_{sig}$, γ, ϰ	240

**Table 6:** The total number of shared keys that should be stored in each node.

Scheme	Number of Shared Keys
Our Proposal	1
[57]	1
[55]	2
[32]	3
[56]	2

**Table 7:** Notation used in computational cost analysis.

Metric	Description
TM	The computing time of the modular multiplication operation.
TA	The computing time of the modular addition operation (negligible).
TIN	The computing time of the modular inversion operation.
TEM	The computing time of the elliptic curve multiplication operation.
TEA	The computing time of the elliptic curve addition operation.

**Table 8:** Computation cost for signature generation and verification.

Schemes	Signing Cost	Verification Cost	Node Cost	Server Cost
Our scheme	TM + TEM + TIN = 41.6 TM	2TM + 2TEM + TIN + TEA = 71.72 TM	41.6 TM (once)	71.72 TM*n
[58]	TM + TEM + TIN = 41.6 TM	2TM + 2TEM + TIN + TEA = 71.72 TM	41.6 TM*n	71.72 TM*n
[60]	TM + TEM = 30 TM	2 TEM + TEA = 58.12 TM	(30 TM + 58.12 TM)*n	(30 TM + 58.12 TM)*n
[59]	TM + 2TEM = 59 TM	2TEM + TEA = 58.12 TM	59 TM*n	58.12 TM*n

**Figure A1:**
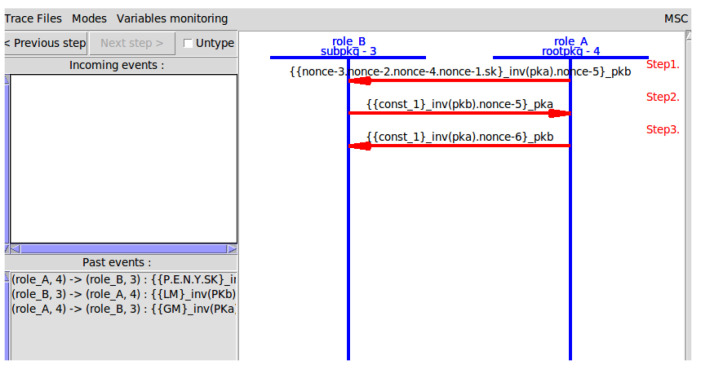
The simulation for the Initialization Phase between RootPKG and $Sub_{PKG}$.

**Figure A2:**
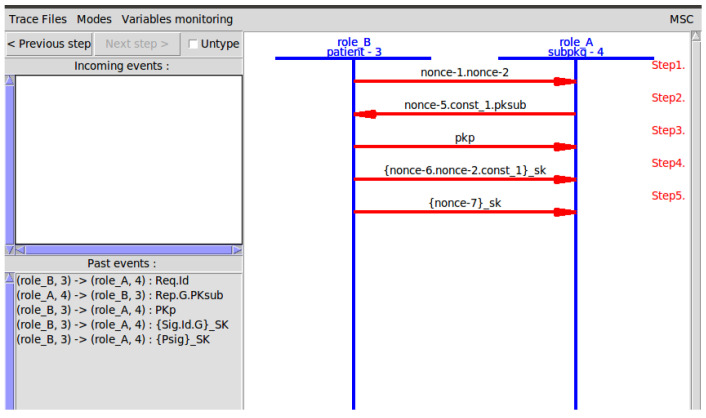
The simulation for Patient Registration Phase between $Sub_{PKG}$ and patient.

**Figure A3:**
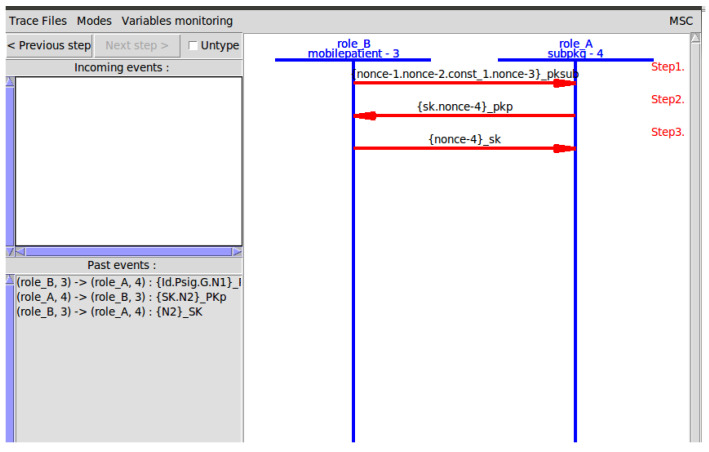
The simulation for Mobile Patient Authentication Phase.

**Figure A4:**
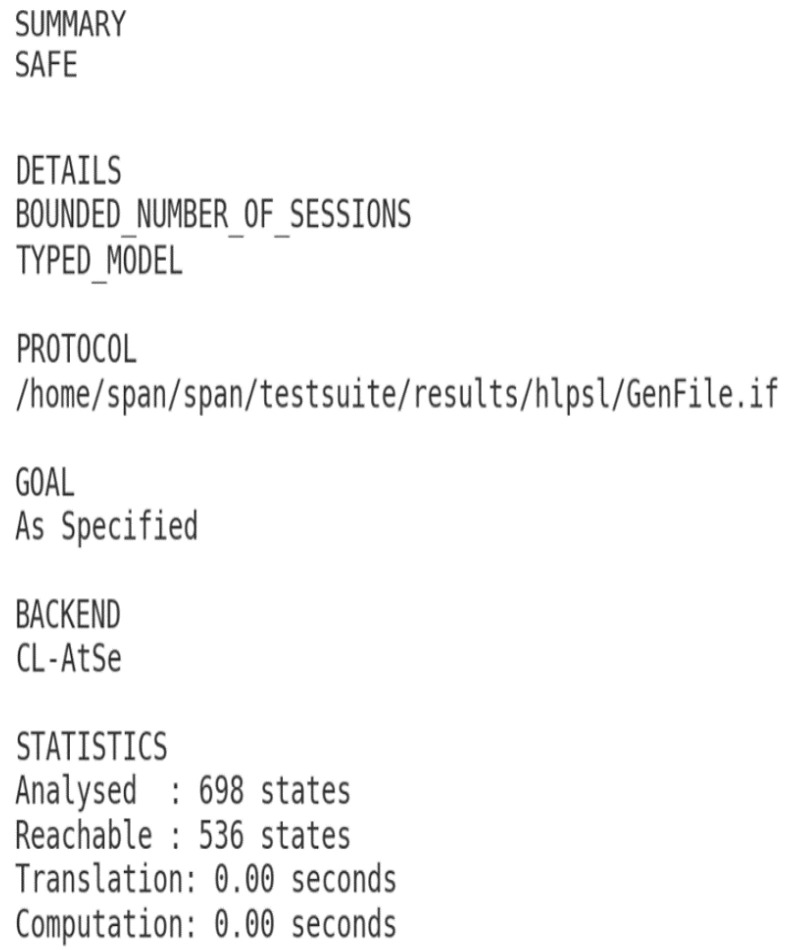
The result for the Initialization Phase between $Root_{PKG}$ and $Sub_{PKG}$ Protocol.

**Figure A5:**
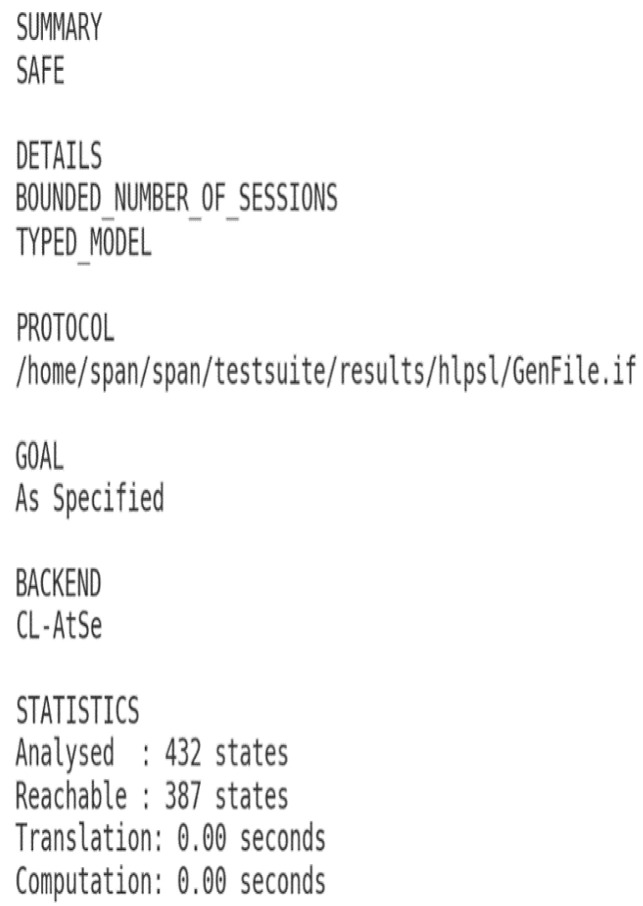
The result for the Patient Registration Phase between $Sub_{PKG}$ and patient Protocol.

**Figure A6:**
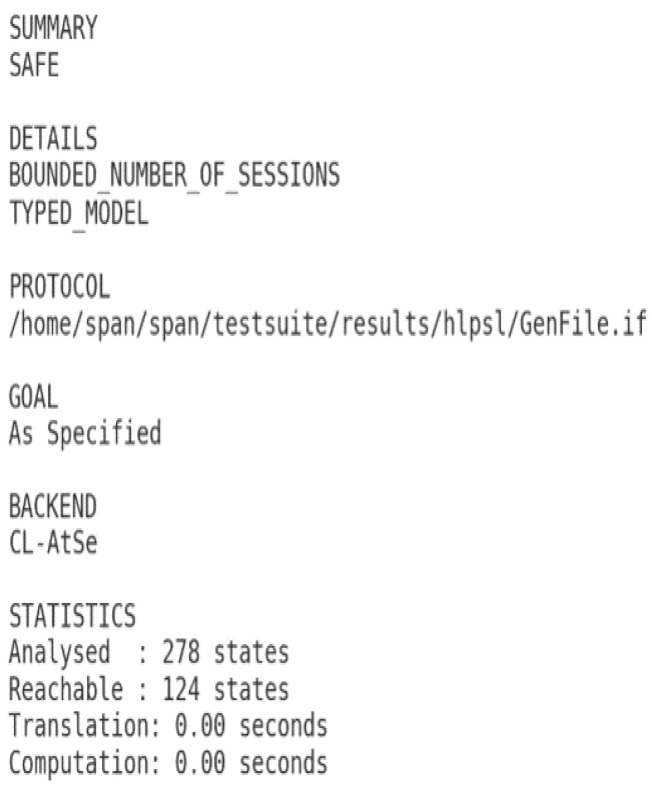
The result for Mobile Patient Authentication Phase Protocol.

## Data Availability

Data are contained within the article.

## Appendix A

### Appendix A.1. Elliptic Curve Diffie–Hellman

Elliptic Curve Diffie–Hellman (ECDH) is a cryptographic key exchange protocol that is widely used to establish a secure communication channel between two parties over an insecure network [44]. It is an extension of the original Diffie–Hellman key exchange protocol, leveraging the mathematical properties of elliptic curves to provide strong security with relatively small key sizes and efficient computations. ECDH is particularly important in modern cryptography as it forms the basis for secure communication in various applications, including secure messaging, secure web browsing (HTTPS), and more.

The elliptic curve process can be summarized by the following [62]:

- Elliptic Curves: At the heart of ECDH is the use of elliptic curves over finite fields. An elliptic curve is a mathematical structure defined by Equation (A1).
  (A1) $$y^{2}=x^{3}+ax+b$$
  where *a* and *b* are constants. The curve is defined over a finite field, which means all calculations are performed modulo a prime number *p*.
- Key Generation: Each party involved in the key exchange process generates its own elliptic curve key pair. This consists of a private key (a randomly chosen number) and a corresponding public key (calculated by multiplying the base point of the curve by the private key).
- Key Exchange: When two parties want to establish a shared secret key, they exchange their public keys over an insecure communication channel.
- Shared Secret Calculation: Each party uses their private key and the received public key to independently compute a shared secret point on the elliptic curve. The magic of elliptic curve mathematics ensures that these independently calculated shared secrets are equal.
- Shared Secret Derivation: The shared secret point is then used as an input to a key derivation function (KDF) to produce a shared secret key. This shared secret can be used for the symmetric encryption and decryption of data between the two parties.

Elliptic Curve Diffie–Hellman (ECDH) offers several distinct advantages when compared to the traditional Diffie–Hellman key exchange. Firstly, ECDH provides robust security despite employing relatively compact key sizes, resulting in efficient computations and reduced bandwidth usage [63]. Moreover, it boasts resilience against quantum attacks, positioning it as a promising candidate for post-quantum cryptography. This protocol excels in terms of efficiency due to the superior performance of elliptic curve operations compared to the conventional modular exponentiation operations used in standard Diffie–Hellman. The compact nature of ECDH keys, considerably shorter than their RSA or DSA counterparts of equivalent strength, renders them ideal for resource-constrained devices and systems. Lastly, ECDH enjoys widespread adoption across modern cryptography standards and protocols, including TLS for secure web browsing, PGP for secure email communication, and numerous others, affirming its significance in ensuring secure data exchange over insecure networks.

### Appendix A.3. BAN Logic

**BAN Logic Notations that are used in this study:**

1. P∣≡ X: P believes X. This means that P considers X to be true and acts accordingly.
2. P ◃ X: P sees X, i.e., when P receive a message contains X, then P sees X.
3. P ∣∼ X: P said X.
4. P ‖∼: P recently said X.
5. P⇒ X: P has jurisdiction over X or P controls X.
6. #(X): The formula X is fresh. This means that X has not been sent in a message at any time before the current run of the protocol.
7. xk: This means that formula X is encrypted using the key k.
8. x $k^{-1}$: This represents that formula X is encrypted using the inverse key of k, i.e., if k is a public key, then $k^{-1}$ is the private key that corresponds to k.
9. PK (k, P): k is a public key for P and there exists a unique key that corresponds to k.
10. II(P): P has a private key that is known only to P.
11. σ(X, P): X is signed by P’s private key.
12. A ${⟷}^{k}$ B , K is a shared key between only A and B.

**Initialization Phase:** Assume that $Sub_{i}$ is $Sub_{PKGi}$, $Root_{i}$ is Root PKGi, PK$Root_{i}$ is the public key of $Root_{i}$, PK$Sub_{i}$ is the public key of the $Sub_{i}$, Pr$Root_{i}$ is the private key of $Root_{i}$, Pr$Sub_{i}$ is the private key of $Sub_{i}$, $M_{1}$ is the first message between $Root_{i}$ and $Sub_{i}$, $M_{2}$ is the second message sent from $Sub_{i}$ to $Root_{i}$, and $M_{3}$ is the third message sent from $Root_{i}$ to $Sub_{i}$.

**Idealized messages:** The following are the idealized messages for this phase based on Figure 3.

- $M_{1}$: $Root_{i}$→$Sub_{i}$: {{P, $N_{1}$, ϵ, η,γ} $Pr_{Root_{i}}$} $PK_{Sub_{i}}$.
- $M_{2}$: $Sub_{i}$→$Root_{i}$:{{LM, $N_{1}$, $N_{2}$} $Pr_{Sub_{i}}$} $PK_{Root_{i}}$.
- $M_{3}$: $Root_{i}$→$Sub_{i}$ {{GM, $N_{2}$,$N_{3}$} $Pr_{Root_{i}}$}$PK_{Sub_{i}}$.

**Assumptions:**

- A1: ${\mathrm{Root}}_{i}\;|\equiv \;\mathrm{PK}\left({\mathrm{Sub}}_{i},{\mathrm{PK}}_{{\mathrm{Sub}}_{i}}\right)$ means that Root PKG Root_*i*_ believes that $PK_{Sub_{i}}$ is the public key of the Sub-$PKG_{i}$ Sub_*i*_.
- A2: ${\mathrm{Root}}_{i}\;|\equiv \;\mathrm{II}\left({\mathrm{Sub}}_{i}\right)$ means that Root PKG Root_*i*_ believes that the Sub_*i*_ has a private key that corresponds to its public key.
- A3: ${\mathrm{Sub}}_{i}\;|\equiv \;\mathrm{PK}\left({\mathrm{Root}}_{i},{\mathrm{PK}}_{{\mathrm{Root}}_{i}}\right)$ means that Sub_*i*_ believes that $PK_{Root_{i}}$ is the public key of Root_*i*_.
- A4: ${\mathrm{Sub}}_{i}\;|\equiv \;\mathrm{II}\left({\mathrm{Root}}_{i}\right)$ means that Sub_*i*_ believes that Root_*i*_ has a private key that corresponds to its public key.
- A5: ${\mathrm{Root}}_{i}\;|\equiv \;{\mathrm{Sub}}_{i}\;\Rightarrow \left(M_{2},\mathrm{LM}\right)$ means that Root PKG Root_*i*_ believes that Sub_*i*_ controls the generation of message $M_{2}$ and local model LM.
- A6: ${\mathrm{Sub}}_{i}\;|\equiv \;{\mathrm{Root}}_{i}\;\Rightarrow \left(M_{1},P\right)$ means that Sub_*i*_ believes that Root_*i*_ controls the generation of message $M_{1}$ and the parameters *P*.
- A7: ${\mathrm{Sub}}_{i}\;|\equiv \;\#{\left(M_{1}\right)}^{\prime }$ means that Sub_*i*_ believes that part of message $M_{1}$ is fresh and has not been sent previously.
- A8: ${\mathrm{Root}}_{i}\;|\equiv \;\#{\left(M_{2}\right)}^{\prime }$ means that Root PKG Root_*i*_ believes that part of message $M_{2}$ is fresh and has not been sent previously.
- A9: ${\mathrm{Sub}}_{i}\;|\equiv \;\#{\left(M_{3}\right)}^{\prime }$ means that Sub_*i*_ believes that part of message $M_{3}$ is fresh and has not been sent previously.
- A10: ${\mathrm{Sub}}_{i}\;|\equiv \;{\mathrm{Root}}_{i}\;\Rightarrow \left(M_{3},\mathrm{GM}\right)$ means that Sub_*i*_ believes that Root_*i*_ controls the generation of message $M_{3}$ and the global model GM.

**Goals:** The goal of the Initialization Phase is to guarantee the mutual authentication between Root_*i*_ and Sub_*i*_. Moreover, Root_*i*_ must trust the LM and believe that it is true, and Sub_*i*_ must trust the GM and believe that it is true. The following are the goals to be achieved in this phase:

- G1: ${\mathrm{Sub}}_{i}\;\left|\equiv \;{\mathrm{Root}}_{i}\;\right|∼M_{1}$ means that Sub_*i*_ should believe that Root_*i*_ has said message $M_{1}$. So, Sub_*i*_ authenticates Root_*i*_.
- G2: ${\mathrm{Sub}}_{i}\;|\equiv M_{1}$ means that Sub_*i*_ should believe message $M_{1}$, which includes the parameters, and consider it true.
- G3: ${\mathrm{Sub}}_{i}\;|\equiv \#M_{1}$ means that Sub_*i*_ should believe that message $M_{1}$ is fresh and not sent before.
- G4: ${\mathrm{Root}}_{i}\;\left|\equiv \;{\mathrm{Sub}}_{i}\;\right|∼M_{2}$ means that the Root PKG Root_*i*_ should believe that Sub_*i*_ has said message $M_{2}$, which includes the local model LM. So, Root_*i*_ authenticates the Sub_*i*_.
- G5: ${\mathrm{Root}}_{i}\;|\equiv M_{2}$ means that the Root PKG Root_*i*_ should believe that message $M_{2}$, which includes the LM is true.
- G6: ${\mathrm{Root}}_{i}\;|\equiv \#M_{2}$ means that the Root PKG Root_*i*_ should believe that message $M_{2}$ is fresh and not sent before.
- G7: ${\mathrm{Sub}}_{i}\;\left|\equiv \;{\mathrm{Root}}_{i}\;\right|∼M_{3}$ means that Sub_*i*_ should believe that Root_*i*_ has said message $M_{3}$.
- G8: ${\mathrm{Sub}}_{i}\;|\equiv M_{3}$ means that Sub_*i*_ should believe message $M_{3}$, which includes the global model GM and consider it true.
- G9: ${\mathrm{Sub}}_{i}\;|\equiv \#M_{3}$ means that Sub_*i*_ should believe that message $M_{3}$ is fresh and not sent before.

**Analysis:**

**Goals G1, G2, and G3:** G1 is achieved via applying the following signing rule on A3, A4, and $M_{1}$. That is, if ${\mathrm{Sub}}_{i}$ receives the signed message $M_{1}$ that is signed using ${\mathrm{PK}}_{{\mathrm{Root}}_{i}}^{-1}$ from ${\mathrm{Root}}_{i}$, and ${\mathrm{Sub}}_{i}$ believes that ${\mathrm{PK}}_{{\mathrm{Root}}_{i}}$ is the public key of ${\mathrm{Root}}_{i}$ and that ${\mathrm{Root}}_{i}$ has a private key that corresponds to ${\mathrm{PK}}_{{\mathrm{Root}}_{i}}$ which is ${\mathrm{PK}}_{{\mathrm{Root}}_{i}}^{-1}$, then ${\mathrm{Sub}}_{i}$ should believe that ${\mathrm{Root}}_{i}$ said message $M_{1}$, which includes the parameters *P*, and ${\mathrm{Sub}}_{i}$ authenticates ${\mathrm{Root}}_{i}$.

$$\begin{array}{cc} \; & \left({\mathrm{Sub}}_{i}\;\left|\equiv \;\mathrm{PK}\left({\mathrm{Root}}_{i},{\mathrm{PK}}_{{\mathrm{Root}}_{i}}\right),\;{\mathrm{Sub}}_{i}\;\right|\equiv \mathrm{II}\left({\mathrm{Root}}_{i}\right),\right.\\ \; & \left.\;{\mathrm{Sub}}_{i}\;▹\;\sigma \left(M_{1},{\mathrm{Root}}_{i}\right)\right)/\left({\mathrm{Sub}}_{i}\;\left|\equiv \;{\mathrm{Root}}_{i}\;\right|∼M_{1}\right),\;G1\;\mathrm{is}\;\mathrm{achieved}. \end{array}$$

G3 is achieved by applying the below freshness rule to A7. That is, if ${\mathrm{Sub}}_{i}$ believes that part of message $M_{1}$ is fresh (which is $N_{1}$), then message $M_{1}$ is fresh.

$$\left({\mathrm{Sub}}_{i}\;|\equiv \;\#{\left(M_{1}\right)}^{\prime }\right)/\left({\mathrm{Sub}}_{i}\;|\equiv \;\#M_{1}\right),\;\mathrm{so}\;G3\;\mathrm{is}\;\mathrm{achieved}.$$

G2 is achieved if the following rules are applied. From G1 and G3, we can infer that ${\mathrm{Sub}}_{i}$ believes that ${\mathrm{Root}}_{i}$ believes message $M_{1}$; then, the control rule is applied to A6. Specifically, if ${\mathrm{Sub}}_{i}$ believes that ${\mathrm{Root}}_{i}$ controls message $M_{1}$, and ${\mathrm{Sub}}_{i}$ believes that ${\mathrm{Root}}_{i}$ believes message $M_{1}$, then ${\mathrm{Sub}}_{i}$ should believe that message $M_{1}$ is true.

$$\left({\mathrm{Sub}}_{i}\;|\equiv \;{\mathrm{Root}}_{i}\;\left|∼M_{1},\;{\mathrm{Sub}}_{i}\;\right|\equiv \;\#M_{1}\right)/\left({\mathrm{Sub}}_{i}\;\left|\equiv \;{\mathrm{Root}}_{i}\;\right|\equiv M_{1}\right),$$

and

$$\begin{array}{c} \left({\mathrm{Sub}}_{i}\;|\equiv \;{\mathrm{Root}}_{i}\;\Rightarrow \left(M_{1},P\right),\;{\mathrm{Sub}}_{i}\;\left|\equiv \;{\mathrm{Root}}_{i}\;\right|\equiv M_{1}\right)/\left({\mathrm{Sub}}_{i}\;|\equiv M_{1}\right),\\ \;G2\;\mathrm{is}\;\mathrm{achieved}. \end{array}$$

**Goals G4, G5, and G6:**

G4 is achieved by applying the below signing rule on A1, A2, and $M_{2}$. That is, if ${\mathrm{Root}}_{i}$ receives the signed message $M_{2}$ that is signed using ${\mathrm{PK}}_{{\mathrm{Sub}}_{i}}^{-1}$ from ${\mathrm{Sub}}_{i}$, and ${\mathrm{Root}}_{i}$ believes that ${\mathrm{PK}}_{{\mathrm{Sub}}_{i}}$ is the public key of ${\mathrm{Sub}}_{i}$ and that ${\mathrm{Sub}}_{i}$ has a private key that corresponds to ${\mathrm{PK}}_{{\mathrm{Sub}}_{i}}$ which is ${\mathrm{PK}}_{{\mathrm{Sub}}_{i}}^{-1}$, then ${\mathrm{Root}}_{i}$ should believe that ${\mathrm{Sub}}_{i}$ said message $M_{2}$, which includes the local model LM, and ${\mathrm{Root}}_{i}$ authenticates ${\mathrm{Sub}}_{i}$.

$$\begin{array}{cc} & \left({\mathrm{Root}}_{i}\;\left|\equiv \;\mathrm{PK}\left({\mathrm{Sub}}_{i},{\mathrm{PK}}_{{\mathrm{Sub}}_{i}}\right),\;{\mathrm{Root}}_{i}\;\right|\equiv \mathrm{II}\left({\mathrm{Sub}}_{i}\right),\right.\\ & \left.\;{\mathrm{Root}}_{i}\;▹\;\sigma \left(M_{2},{\mathrm{Sub}}_{i}\right)\right)/\left({\mathrm{Root}}_{i}\;\left|\equiv \;{\mathrm{Sub}}_{i}\;\right|∼M_{2}\right),\\ & \;G4\;\mathrm{is}\;\mathrm{achieved}. \end{array}$$

G6 is achieved if the below freshness rule on A8 is applied. That is, if ${\mathrm{Root}}_{i}$ believes that part of message $M_{2}$ is fresh (which is $N_{2}$), then message $M_{2}$ is fresh.

$$\left({\mathrm{Root}}_{i}\;|\equiv \;\#{\left(M_{2}\right)}^{\prime }\right)/\left({\mathrm{Root}}_{i}\;|\equiv \;\#M_{2}\right),\;\mathrm{so}\;G6\;\mathrm{is}\;\mathrm{achieved}.$$

G5 is achieved if the following rules are applied. From G4 and G6, we can infer that ${\mathrm{Root}}_{i}$ believes that ${\mathrm{Sub}}_{i}$ believes message $M_{2}$; then, the control rule is applied to A5. Specifically, if ${\mathrm{Root}}_{i}$ believes that ${\mathrm{Sub}}_{i}$ controls message $M_{2}$, and ${\mathrm{Root}}_{i}$ believes that ${\mathrm{Sub}}_{i}$ believes message $M_{2}$, then ${\mathrm{Root}}_{i}$ should believe that message $M_{2}$ is true.

$$\left({\mathrm{Root}}_{i}\;|\equiv \;{\mathrm{Sub}}_{i}\;\left|∼M_{2},\;{\mathrm{Root}}_{i}\;\right|\equiv \;\#M_{2}\right)/\left({\mathrm{Root}}_{i}\;\left|\equiv \;{\mathrm{Sub}}_{i}\;\right|\equiv M_{2}\right),$$

and

$$\begin{array}{cc} \; & \left({\mathrm{Root}}_{i}\;|\equiv \;{\mathrm{Sub}}_{i}\;\Rightarrow \left(M_{2},\mathrm{LM}\right),\right.\\ \; & \left.\;{\mathrm{Root}}_{i}\;\left|\equiv \;{\mathrm{Sub}}_{i}\;\right|\equiv M_{2}\right)/\left({\mathrm{Root}}_{i}\;|\equiv M_{2}\right),\;G5\;\mathrm{is}\;\mathrm{achieved}. \end{array}$$

**Goals G7, G8, and G9:**

G7 is achieved by applying the following signing rule on A3, A4, and $M_{3}$. That is, if ${\mathrm{Sub}}_{i}$ receives the signed message $M_{3}$ that is signed using ${\mathrm{PK}}_{{\mathrm{Root}}_{i}}^{-1}$ from ${\mathrm{Root}}_{i}$, and ${\mathrm{Sub}}_{i}$ believes that ${\mathrm{PK}}_{{\mathrm{Root}}_{i}}$ is the public key of ${\mathrm{Root}}_{i}$ and that ${\mathrm{Root}}_{i}$ has a private key that corresponds to ${\mathrm{PK}}_{{\mathrm{Root}}_{i}}$ which is ${\mathrm{PK}}_{{\mathrm{Root}}_{i}}^{-1}$, then ${\mathrm{Sub}}_{i}$ should believe that ${\mathrm{Root}}_{i}$ said message $M_{3}$, which includes the global model GM.

$$\begin{array}{cc} \; & \left({\mathrm{Sub}}_{i}\;\left|\equiv \;\mathrm{PK}\left({\mathrm{Root}}_{i},{\mathrm{PK}}_{{\mathrm{Root}}_{i}}\right),\;{\mathrm{Sub}}_{i}\;\right|\equiv \mathrm{II}\left({\mathrm{Root}}_{i}\right),\right.\\ \; & \left.\;{\mathrm{Sub}}_{i}\;▹\;\sigma \left(M_{3},{\mathrm{Root}}_{i}\right)\right)/\left({\mathrm{Sub}}_{i}\;\left|\equiv \;{\mathrm{Root}}_{i}\;\right|∼M_{3}\right),\\ \; & \;G7\;\mathrm{is}\;\mathrm{achieved}. \end{array}$$

By applying the following freshness rule to A9, G9 is achieved. That is, if ${\mathrm{Sub}}_{i}$ believes that part of message $M_{3}$ is fresh (which is $N_{3}$), then message $M_{3}$ is fresh.

$$\left({\mathrm{Sub}}_{i}\;|\equiv \;\#{\left(M_{3}\right)}^{\prime }\right)/\left({\mathrm{Sub}}_{i}\;|\equiv \;\#M_{3}\right),\;\mathrm{so}\;G9\;\mathrm{is}\;\mathrm{achieved}.$$

G8 is achieved via applying the following rules. From G7 and G9, we can infer that ${\mathrm{Sub}}_{i}$ believes that ${\mathrm{Root}}_{i}$ believes message $M_{3}$; then, the control rule is applied to A10. Specifically, if ${\mathrm{Sub}}_{i}$ believes that ${\mathrm{Root}}_{i}$ controls message $M_{3}$, and ${\mathrm{Sub}}_{i}$ believes that ${\mathrm{Root}}_{i}$ believes message $M_{3}$, then ${\mathrm{Sub}}_{i}$ should believe that message $M_{3}$ is true.

$$\left({\mathrm{Sub}}_{i}\;|\equiv \;{\mathrm{Root}}_{i}\;\left|∼M_{3},\;{\mathrm{Sub}}_{i}\;\right|\equiv \;\#M_{3}\right)/\left({\mathrm{Sub}}_{i}\;\left|\equiv \;{\mathrm{Root}}_{i}\;\right|\equiv M_{3}\right),$$

and

$$\begin{array}{cc} \; & \left({\mathrm{Sub}}_{i}\;|\equiv \;{\mathrm{Root}}_{i}\;\Rightarrow \left(M_{3},\mathrm{GM}\right),\;{\mathrm{Sub}}_{i}\;\left|\equiv \;{\mathrm{Root}}_{i}\;\right|\equiv M_{3}\right)/\\ \; & \left({\mathrm{Sub}}_{i}\;|\equiv M_{3}\right),\;G8\;\mathrm{is}\;\mathrm{achieved}. \end{array}$$

**Patient Registration Phase**

In this section, assume the following:

- ${\mathrm{Sub}}_{i}$ is Sub-PKG*i*
- ${\mathrm{PK}}_{{\mathrm{Sub}}_{i}}$ is the public key of ${\mathrm{Sub}}_{i}$
- ${\mathrm{Pr}}_{{\mathrm{Sub}}_{i}}$ is the private key of ${\mathrm{Sub}}_{i}$
- ${\mathrm{PK}}_{{\mathrm{Patient}}_{i}}$ is the public key of ${\mathrm{Patient}}_{i}$
- ${\mathrm{Pr}}_{{\mathrm{Patient}}_{i}}$ is the private key of ${\mathrm{Patient}}_{i}$
- ${\mathrm{SK}}_{{\mathrm{Sub}}_{i}}$ is the session key between ${\mathrm{Sub}}_{i}$ and ${\mathrm{Patient}}_{i}$
- ${\mathrm{PKDH}}_{{\mathrm{Patient}}_{i}}$ is the public component of ${\mathrm{Patient}}_{i}$
- ${\mathrm{PKDH}}_{{\mathrm{Sub}}_{i}}$ is the public component of ${\mathrm{Sub}}_{i}$
- ${\mathrm{DC}}_{{\mathrm{Sub}}_{i}}$ is the digital certificate for ${\mathrm{Sub}}_{i}$
- ${\mathrm{DC}}_{{\mathrm{Patient}}_{i}}$ is the digital certificate of ${\mathrm{Patient}}_{i}$
- M1 is the first message in this phase between ${\mathrm{Patient}}_{i}$ and ${\mathrm{Sub}}_{i}$
- M2 is the second message in this phase sent from ${\mathrm{Sub}}_{i}$ to ${\mathrm{Patient}}_{i}$
- M3 is the third message in this phase sent from ${\mathrm{Patient}}_{i}$ to ${\mathrm{Sub}}_{i}$
- M4 is the fourth message in this phase sent from ${\mathrm{Sub}}_{i}$ to ${\mathrm{Patient}}_{i}$
- M5 is the fifth message sent from ${\mathrm{Patient}}_{i}$ to ${\mathrm{Sub}}_{i}$

**Idealized messages:**

The following are the idealized messages for this phase based on Figure 4:

- M1: ${\mathrm{Patient}}_{i}\to {\mathrm{Sub}}_{i}$: ID||N1
- M2: ${\mathrm{Sub}}_{i}\to {\mathrm{Patient}}_{i}$: ${\mathrm{DC}}_{{\mathrm{Sub}}_{i}}\left|\right|\left\{G,N1,N2,{\mathrm{PKDH}}_{{\mathrm{Sub}}_{i}}\right\}{\mathrm{Pr}}_{{\mathrm{Sub}}_{i}}$
- M3: ${\mathrm{Patient}}_{i}\to {\mathrm{Sub}}_{i}$: ${\mathrm{DC}}_{{\mathrm{Patient}}_{i}}\left|\right|\left\{{\mathrm{PKDH}}_{{\mathrm{Patient}}_{i}},N2\right\}{\mathrm{Pr}}_{{\mathrm{Patient}}_{i}}$
- M4: ${\mathrm{Sub}}_{i}\to {\mathrm{Patient}}_{i}$: $\left\{\sigma_{\mathrm{GS}},\Upsilon ,\gamma ,\mathrm{Idg},N3\right\}{\mathrm{SK}}_{{\mathrm{Sub}}_{i}}$
- M5: ${\mathrm{Patient}}_{i}\to {\mathrm{Sub}}_{i}$: $\left\{\mathrm{Psig},N3\right\}{\mathrm{SK}}_{{\mathrm{Sub}}_{i}}$

**Assumptions:**

- A1: ${\mathrm{Sub}}_{i}|\equiv {\mathrm{Sub}}_{i}□\left(↔\left({\mathrm{SK}}_{{\mathrm{Sub}}_{i}}\right)\right){\mathrm{Patient}}_{i}$ means that ${\mathrm{Sub}}_{i}$ believes that ${\mathrm{SK}}_{{\mathrm{Sub}}_{i}}$ is a key shared between ${\mathrm{Patient}}_{i}$ and ${\mathrm{Sub}}_{i}$
- A2: ${\mathrm{Patient}}_{i}|\equiv {\mathrm{Sub}}_{i}□\left(↔\left({\mathrm{SK}}_{{\mathrm{Sub}}_{i}}\right)\right){\mathrm{Patient}}_{i}$ means that ${\mathrm{Patient}}_{i}$ believes that ${\mathrm{SK}}_{{\mathrm{Sub}}_{i}}$ is a key shared between ${\mathrm{Patient}}_{i}$ and ${\mathrm{Sub}}_{i}$

**Goals:**

This phase aims to achieve mutual authentication between the Sub-PKG and the patient, and to generate the session key shared between them. Therefore, both ${\mathrm{Patient}}_{i}$ and ${\mathrm{Sub}}_{i}$ should believe and trust that the session key is true. Note that public Diffie–Hellman components ${\mathrm{PKDH}}_{{\mathrm{Patient}}_{i}}$ and ${\mathrm{PKDH}}_{{\mathrm{Sub}}_{i}}$ are shared in signed messages (M2 and M3) which means the authenticity of these components can be proved in the same way as performed in the previous phase. In this section, we will focus on the trust of the generated session key. The following are the goals to be achieved:

- G1: ${\mathrm{Patient}}_{i}\left|\equiv {\mathrm{Sub}}_{i}\right|∼M_{4}$ means that ${\mathrm{Patient}}_{i}$ should believe that ${\mathrm{Sub}}_{i}$ has sent message $M_{4}$, which includes N3. So, ${\mathrm{Patient}}_{i}$ authenticates ${\mathrm{Sub}}_{i}$.
- G2: ${\mathrm{Sub}}_{i}\left|\equiv {\mathrm{Patient}}_{i}\right|∼M_{5}$ means that ${\mathrm{Sub}}_{i}$ should believe that ${\mathrm{Patient}}_{i}$ has sent message $M_{5}$ which includes the same nonce N3. So, ${\mathrm{Sub}}_{i}$ authenticates ${\mathrm{Patient}}_{i}$.

**Analysis:**

G1 is achieved via applying the below symmetric rule on A2 and $M_{4}$. That is, if ${\mathrm{Patient}}_{i}$ receives message $M_{4}$ that is encrypted using ${\mathrm{SK}}_{{\mathrm{Sub}}_{i}}$ from ${\mathrm{Sub}}_{i}$, and ${\mathrm{Patient}}_{i}$ believes that ${\mathrm{SK}}_{{\mathrm{Sub}}_{i}}$ is the shared key between ${\mathrm{Patient}}_{i}$ and ${\mathrm{Sub}}_{i}$, then ${\mathrm{Patient}}_{i}$ should believe that ${\mathrm{Sub}}_{i}$ sent message $M_{4}$ and so ${\mathrm{Sub}}_{i}$ is authenticated.

$$\begin{array}{cc} \; & \left({\mathrm{Patient}}_{i}⊢\left\{M_{4}\right\}{\mathrm{SK}}_{{\mathrm{Sub}}_{i}},\;{\mathrm{Patient}}_{i}|\equiv {\mathrm{Sub}}_{i}□\left(↔\left({\mathrm{SK}}_{{\mathrm{Sub}}_{i}}\right)\right){\mathrm{Patient}}_{i}\right)\\ \; & \;/\left({\mathrm{Patient}}_{i}\left|\equiv {\mathrm{Sub}}_{i}\right|∼M_{4}\right),\;G1\;\mathrm{is}\;\mathrm{achieved}. \end{array}$$

Similarly, G2 is achieved if the below symmetric rule is applied to A1 and $M_{5}$. That is, if ${\mathrm{Sub}}_{i}$ receives message $M_{5}$ that is encrypted using ${\mathrm{SK}}_{{\mathrm{Sub}}_{i}}$ from ${\mathrm{Patient}}_{i}$, and ${\mathrm{Sub}}_{i}$ believes that ${\mathrm{SK}}_{{\mathrm{Sub}}_{i}}$ is the shared key between ${\mathrm{Patient}}_{i}$ and ${\mathrm{Sub}}_{i}$, then ${\mathrm{Sub}}_{i}$ should believe that ${\mathrm{Patient}}_{i}$ sent message $M_{5}$ and so ${\mathrm{Patient}}_{i}$ is authenticated.

$$\begin{array}{cc} \; & \left({\mathrm{Sub}}_{i}⊢\left\{M_{5}\right\}{\mathrm{SK}}_{{\mathrm{Sub}}_{i}},\;{\mathrm{Sub}}_{i}|\equiv {\mathrm{Sub}}_{i}□\left(↔\left({\mathrm{SK}}_{{\mathrm{Sub}}_{i}}\right)\right){\mathrm{Patient}}_{i}\right)\\ \; & \;/\left({\mathrm{Sub}}_{i}\left|\equiv {\mathrm{Patient}}_{i}\right|∼M_{5}\right),\;G2\;\mathrm{is}\;\mathrm{achieved}. \end{array}$$

**Patient Authentication Phase**

Assume that ${\mathrm{Sub}}_{i}$ is Sub-PKG_*i*_, ${\mathrm{PK}}_{{\mathrm{Sub}}_{i}}$ is the public key of ${\mathrm{Sub}}_{i}$, ${\mathrm{Pr}}_{{\mathrm{Sub}}_{i}}$ is the private key of ${\mathrm{Sub}}_{i}$, ${\mathrm{PK}}_{{\mathrm{Patient}}_{i}}$ is the public key of ${\mathrm{Patient}}_{i}$, ${\mathrm{Pr}}_{{\mathrm{Patient}}_{i}}$ is the private key of ${\mathrm{Patient}}_{i}$, and SK is the session key between ${\mathrm{Sub}}_{i}$ and ${\mathrm{Patient}}_{i}$.

${\mathrm{DC}}_{{\mathrm{Sub}}_{i}}$ is the digital certificate for ${\mathrm{Sub}}_{i}$, ${\mathrm{DC}}_{{\mathrm{Patient}}_{i}}$ is the digital certificate for ${\mathrm{Patient}}_{i}$, $M_{1}$ is the first message between ${\mathrm{Patient}}_{i}$ and ${\mathrm{Sub}}_{i}$, $M_{2}$ is the second message sent from ${\mathrm{Sub}}_{i}$ to ${\mathrm{Patient}}_{i}$, and $M_{3}$ is the third message in this phase sent from ${\mathrm{Patient}}_{i}$ to ${\mathrm{Sub}}_{i}$.

**Idealized messages:** The below are the idealized messages based on Figure 4.

- $M_{1}$:
  $$\begin{array}{cc} & {\mathrm{Patient}}_{i}\to {\mathrm{Sub}}_{i}:\{{\mathrm{DC}}_{{\mathrm{Patient}}_{i}}||\left\{\mathrm{Idg},\mathrm{Psig},X,N1\right\}{\mathrm{Pr}}_{{\mathrm{Patient}}_{i}}\}{\mathrm{PK}}_{{\mathrm{Sub}}_{i}} \end{array}$$
- $M_{2}$: ${\mathrm{Sub}}_{i}\to {\mathrm{Patient}}_{i}$: $\left\{\left\{\mathrm{SK},N1,N2\right\}{\mathrm{Pr}}_{{\mathrm{Sub}}_{i}}\right\}{\mathrm{PK}}_{{\mathrm{Patient}}_{i}}$
- $M_{3}$: ${\mathrm{Patient}}_{i}\to {\mathrm{Sub}}_{i}$: {N2}SK

**Assumptions:**

- A1: ${\mathrm{Patient}}_{i}|\equiv \mathrm{PK}\left({\mathrm{Sub}}_{i},{\mathrm{PK}}_{{\mathrm{Sub}}_{i}}\right)$ means that ${\mathrm{Patient}}_{i}$ believes that ${\mathrm{PK}}_{{\mathrm{Sub}}_{i}}$ is the public key of ${\mathrm{Sub}}_{i}$.
- A2: ${\mathrm{Patient}}_{i}|\equiv \mathrm{II}\left({\mathrm{Sub}}_{i}\right)$ means that ${\mathrm{Patient}}_{i}$ believes that ${\mathrm{Sub}}_{i}$ has a private key that corresponds to its public key.
- A3: ${\mathrm{Sub}}_{i}|\equiv \mathrm{PK}\left({\mathrm{Patient}}_{i},{\mathrm{PK}}_{{\mathrm{Patient}}_{i}}\right)$ means that ${\mathrm{Sub}}_{i}$ believes that ${\mathrm{PK}}_{{\mathrm{Patient}}_{i}}$ is the public key of ${\mathrm{Patient}}_{i}$.
- A4: ${\mathrm{Sub}}_{i}|\equiv \mathrm{II}\left({\mathrm{Patient}}_{i}\right)$ means that ${\mathrm{Sub}}_{i}$ believes that ${\mathrm{Patient}}_{i}$ has a private key that corresponds to its public key.
- A5: ${\mathrm{Sub}}_{i}|\equiv \#{\left(M_{1}\right)}^{\prime }$ means that ${\mathrm{Sub}}_{i}$ believes that part of message $M_{1}$ is fresh and has not been sent previously.
- A6: ${\mathrm{Patient}}_{i}|\equiv \#{\left(M_{2}\right)}^{\prime }$ means that ${\mathrm{Patient}}_{i}$ believes that part of message $M_{2}$ is fresh and has not been sent previously.
- A7: ${\mathrm{Sub}}_{i}|\equiv {\mathrm{Patient}}_{i}\Rightarrow \left(M_{1},\mathrm{Psig}\right)$ means that ${\mathrm{Sub}}_{i}$ believes that ${\mathrm{Patient}}_{i}$ controls the generation of message $M_{1}$ which includes Psig.
- A8: ${\mathrm{Patient}}_{i}|\equiv {\mathrm{Sub}}_{i}\Rightarrow \left(M_{2},\mathrm{SK}\right)$ means that ${\mathrm{Patient}}_{i}$ believes that ${\mathrm{Sub}}_{i}$ controls the generation of message $M_{2}$ and the session key SK.
- A9: ${\mathrm{Sub}}_{i}|\equiv {\mathrm{Sub}}_{i}□\left(↔\left(\mathrm{SK}\right)\right){\mathrm{Patient}}_{i}$ means that ${\mathrm{Sub}}_{i}$ believes that SK is a key shared between ${\mathrm{Patient}}_{i}$ and ${\mathrm{Sub}}_{i}$.
- A10: ${\mathrm{Patient}}_{i}|\equiv {\mathrm{Sub}}_{i}□\left(↔\left(\mathrm{SK}\right)\right){\mathrm{Patient}}_{i}$ means that ${\mathrm{Patient}}_{i}$ believes that SK is a key shared between ${\mathrm{Patient}}_{i}$ and ${\mathrm{Sub}}_{i}$.

**Goals:** The goal of this phase is to authenticate the patient and to generate the session key SK shared between them. Therefore, both ${\mathrm{Patient}}_{i}$ and ${\mathrm{Sub}}_{i}$ should believe and trust that the session key is true. The following are the goals to be achieved in this phase.

- G1: ${\mathrm{Sub}}_{i}\left|\equiv {\mathrm{Patient}}_{i}\right|∼M_{1}$ means that ${\mathrm{Sub}}_{i}$ should believe that ${\mathrm{Patient}}_{i}$ has sent message $M_{1}$, which includes the patient’s signature Psig.
- G2: ${\mathrm{Sub}}_{i}|\equiv M_{1}$ means that ${\mathrm{Sub}}_{i}$ should believe message $M_{1}$.
- G3: ${\mathrm{Sub}}_{i}|\equiv \#M_{1}$ means that ${\mathrm{Sub}}_{i}$ should believe that message $M_{1}$ is fresh and has not been sent before.
- G4: ${\mathrm{Patient}}_{i}\left|\equiv {\mathrm{Sub}}_{i}\right|∼M_{2}$ means that ${\mathrm{Patient}}_{i}$ should believe that ${\mathrm{Sub}}_{i}$ has sent message $M_{2}$, which includes the session key SK.
- G5: ${\mathrm{Patient}}_{i}|\equiv M_{2}$ means that ${\mathrm{Patient}}_{i}$ should believe message $M_{2}$ which includes the session key SK.
- G6: ${\mathrm{Patient}}_{i}|\equiv \#M_{2}$ means that ${\mathrm{Patient}}_{i}$ should believe that message $M_{2}$, which includes the session key SK, is fresh and has not been sent before.
- G7: ${\mathrm{Sub}}_{i}\left|\equiv {\mathrm{Patient}}_{i}\right|∼M_{3}$ means that ${\mathrm{Sub}}_{i}$ should believe that ${\mathrm{Patient}}_{i}$ has sent message $M_{3}$, which includes the same nonce N2. So, ${\mathrm{Sub}}_{i}$ authenticates ${\mathrm{Patient}}_{i}$.

**Analysis:**

**Goals G1, G2, and G3:** G1 is achieved via applying the below signing rule to A3, A4, and $M_{1}$. That is, if ${\mathrm{Sub}}_{i}$ receives the signed message $M_{1}$ that is signed using ${\mathrm{PK}}_{{\mathrm{Patient}}_{i}}$ from ${\mathrm{Patient}}_{i}$, and ${\mathrm{Sub}}_{i}$ believes that ${\mathrm{PK}}_{{\mathrm{Patient}}_{i}}$ is the public key of ${\mathrm{Patient}}_{i}$ and that ${\mathrm{Patient}}_{i}$ has a private key corresponding to ${\mathrm{PK}}_{{\mathrm{Patient}}_{i}}$, then ${\mathrm{Sub}}_{i}$ should believe that ${\mathrm{Patient}}_{i}$ sent message $M_{1}$, which includes the patient’s signature Psig.

$$\begin{array}{cc} \; & ({\mathrm{Sub}}_{i}|\equiv \mathrm{PK}\left({\mathrm{Patient}}_{i},{\mathrm{PK}}_{{\mathrm{Patient}}_{i}}\right)),\\ \; & ({\mathrm{Sub}}_{i}|\equiv \mathrm{II}\left({\mathrm{Patient}}_{i}\right)),\\ \; & ({\mathrm{Sub}}_{i}⊢\sigma \left(M_{1},{\mathrm{Patient}}_{i}\right))/\\ \; & ({\mathrm{Sub}}_{i}|\equiv {\mathrm{Patient}}_{i}|∼M_{1}),\;G1\;\mathrm{is}\;\mathrm{achieved}. \end{array}$$

G3 is achieved by applying the below freshness rule to A5. That is, if $Sub_{i}$ believes that part of message M1 is fresh (which is NI), then message M1 is fresh.

$$\left(Sub_{i}\;\equiv \#{\left(M_{1}\right)}^{\prime },\right)/\left(Sub_{i}\;\equiv \#M_{1}\right),\mathrm{so}\;G3\;\mathrm{is}\;\mathrm{achieved}.$$

G2 is achieved via applying the below rules. From G1 and G3, we can infer that $Sub_{i}$ believes that $Patient_{i}$ believes message M1; then, the control rule is applied to A7. Specifically, if $Sub_{i}$ believes that $Patient_{i}$ controls message M1, and $Sub_{i}$ believes that $Patient_{i}$ believes message M1, then $Sub_{i}$ should believe that message M1 is true.

$$\begin{array}{c} \begin{array}{c} (Sub_{i}\equiv Patient_{i}|∼M_{1},\;Sub_{i}\equiv \#M_{1})/\\ (Sub_{i}\equiv Patient_{i}\equiv M_{1}), \end{array}\\ \mathrm{and}\;(Sub_{i}\equiv Patient_{i}\Rightarrow \left(M_{1},Psig\right),\\ Sub_{i}\equiv Patient_{i}\equiv M_{1})/\left(Sub_{i}\equiv M_{1}\right),G2\;\mathrm{is}\;\mathrm{achieved}. \end{array}$$

Goals G4, G5, and G6:

Similarly, G4 is achieved by applying the below signing rule to A1, A2, and M2. That is, if $Patient_{i}$ receives the signed message M2 that is signed using PKsubi-1 from $Sub_{i}$, and $Patient_{i}$ believes that PKsubi is the public key of $Sub_{i}$ and that $Sub_{i}$ has a private key corresponding to PKsubi which is PKsubi-1, then $Patient_{i}$ should believe that $Sub_{i}$ said message M2, which includes the session key SK.

$$\begin{array}{c} (Patient_{i}\equiv PK\left(Sub_{i},PK_{Sub_{i}}\right),\\ \;Patient_{i}\equiv II\left(Sub_{i}\right),\;Patient_{i}⊢\sigma \left(M_{2},Sub_{i}\right))/\\ (Patient_{i}\equiv Sub_{i}|∼M_{2}),G4\;\mathrm{is}\;\mathrm{achieved}. \end{array}$$

G6 is also achieved by applying the following freshness rule to A6. That is, if $Patient_{i}$ believes that part of message M2 is fresh (which is N2), then message M2 is fresh.

$$\left(Patient_{i}\;\equiv \#{\left(M_{2}\right)}^{\prime },\right)/\left(Patient_{i}\;\equiv \#M_{2}\right),\mathrm{so}\;G6\;\mathrm{is}\;\mathrm{achieved}.$$

G5 is achieved via applying the below rules. From G4 and G6, we can infer that $Patient_{i}$ believes that $Sub_{i}$ believes message M2; then, we apply the control rule to A8. Specifically, if $Patient_{i}$ believes that $Sub_{i}$ controls message M2, and $Patient_{i}$ believes that $Sub_{i}$ believes message M2, then $Patient_{i}$ should believe that message M2 is true.

$$\begin{array}{cc} & (Patient_{i}\equiv Sub_{i}|∼M_{2},\;Patient_{i}\equiv \#M_{2})/\\ & \left(Patient_{i}\equiv Sub_{i}\equiv M_{2}\right),\mathrm{and}\;(Patient_{i}\equiv Sub_{i}\Rightarrow \left(M_{2},SK\right),\\ & \;Patient_{i}\equiv Sub_{i}\equiv M_{2})/\left(Patient_{i}\equiv M_{2}\right),G5\;\mathrm{is}\;\mathrm{achieved}. \end{array}$$

Goal G7:

Finally, G7 is achieved by applying the below symmetric rule to A9 and M3. That is, if $Sub_{i}$ receives message M3 that is encrypted using SK from $Patient_{i}$, and $Sub_{i}$ believes that SK is the shared key between $Sub_{i}$ and $Patient_{i}$, then $Sub_{i}$ should believe that $Patient_{i}$ said message M3, which includes the nonce N2, so $Patient_{i}$ is authenticated.

$$\begin{array}{cc} \; & (Sub_{i}⊢\left\{M_{3}\right\}SK,\;Sub_{i}\equiv Sub_{i}□\left(↔\left(SK\right)\right)\;Patient_{i})/\\ \; & (Sub_{i}\equiv Patient_{i}|∼M_{3}),G7\;\mathrm{is}\;\mathrm{achieved}. \end{array}$$
